# The Roles of Direct Recognition by Animal Lectins in Antiviral Immunity and Viral Pathogenesis

**DOI:** 10.3390/molecules20022272

**Published:** 2015-01-29

**Authors:** Yang Liu, Jianying Liu, Xiaojing Pang, Tao Liu, Zhijie Ning, Gong Cheng

**Affiliations:** 1 Department of Basic Medical Sciences, School of Medicine, Tsinghua University, Beijing 100084, China; E-Mails: liu-y-13@mails.tsinghua.edu.cn (Y.L.); liujianying12@mails.tsinghua.edu.cn (J.L.); pangxj2471@mail.tsinghua.edu.cn (X.P.); 2 Collaborative Innovation Center for Diagnosis and Treatment of Infectious Diseases, Hangzhou 310003, China; 3 School of Life Science, Tsinghua University, Beijing 100084, China; 4 Center for Reproductive Medicine, Tai’an Central Hospital, Tai’an 271000, China; E-Mail: liutao770404@163.com; 5 Ji’nan Infectious Diseases Hospital, Ji’nan 250021, China; E-Mail: nzjbear@126.com; 6 Tsinghua-Peking Joint Center for Life Sciences, Tsinghua University, Beijing 100084, China

**Keywords:** lectin, virus, direct interaction, antiviral immunity, viral pathogenesis

## Abstract

Lectins are a group of proteins with carbohydrate recognition activity. Lectins are categorized into many families based on their different cellular locations as well as their specificities for a variety of carbohydrate structures due to the features of their carbohydrate recognition domain (CRD) modules. Many studies have indicated that the direct recognition of particular oligosaccharides on viral components by lectins is important for interactions between hosts and viruses. Herein, we aim to globally review the roles of this recognition by animal lectins in antiviral immune responses and viral pathogenesis. The different classes of mammalian lectins can either recognize carbohydrates to activate host immunity for viral elimination or can exploit those carbohydrates as susceptibility factors to facilitate viral entry, replication or assembly. Additionally, some arthropod C-type lectins were recently identified as key susceptibility factors that directly interact with multiple viruses and then facilitate infection. Summarization of the pleiotropic roles of direct viral recognition by animal lectins will benefit our understanding of host-virus interactions and could provide insight into the role of lectins in antiviral drug and vaccine development.

## 1. Introduction

Lectins, a highly diverse group of proteins that recognize carbohydrates, have been demonstrated to play a vital role in numerous life processes and to be critical for several viral infections and pathogeneses in a variety of organisms [1,2]. Based on their conserved structure of sequence motifs for sugar binding and carbohydrate specificities, lectins have been categorized into many families conventionally designated as calnexin, C-type, L-type, P-type/mannose-6-phosphate receptors (MPRs), I-type/siglecs, M-type, F-type (absent in mammals), R-type, F-box, chitinase-like lectins, galectins and intelectins (Table 1) [3]. The features of carbohydrate recognition domains (CRDs), such as structure peculiarity, carbohydrate binding selectivity and geometrical arrangement of multiple CRDs, determine the different properties of lectins [2,3,4]. Furthermore, the diversity of locations and functions indicates the importance of lectins in the basic life processes of organisms.

**Table 1:** The classification of animal lectins.

Lectin	Saccharide Specificity	Core motif	Location	Direct Interaction to Viral Components
C-type lectin	Variable	C-type sequence motif	Extracellular, Cell membrane	Yes
Galectin	β-Galactosides	S-type sequence motif	Extracellular, Cytoplasm	Yes
Calnexin	Glc_1_Man_9_	Calnexin sequence motif	ER	Yes
P-type lectin	Mannose-6-P, others	P-type sequence motif	Cell membrane, Endosome	Yes
L-type lectin	Variable	L-type sequence motif	ER, ERGIC, Golgi	Yes
I-type lectin	Sialic acid, variable	Ig-like domains	Cell membrane	No
M-type lectin	Man_8_	M-type sequence motif	ER	No
F-type lectin	L-fucose	F-type sequence motif	Extracellular	No
R-type lectin	Variable	R-type sequence motif	Extracellular, Cell membrane	No
F-box lectin	GlcNAc_2_	F-box sequence motif	Cytoplasm	No
Chitinase-like lectin	Chito-oligosaccharides	TIM (Triose-phosphate isomerase) barrel-like structure	Extracellular	No
Intelectin	Gal, glactofuranose, pentoses	Intelectin sequence motif	Extracellular, Cell membrane	No

Notes: ER: Endoplasmic reticulum; ERGIC: ER-Golgi intermediate compartment.

**Figure 1:**
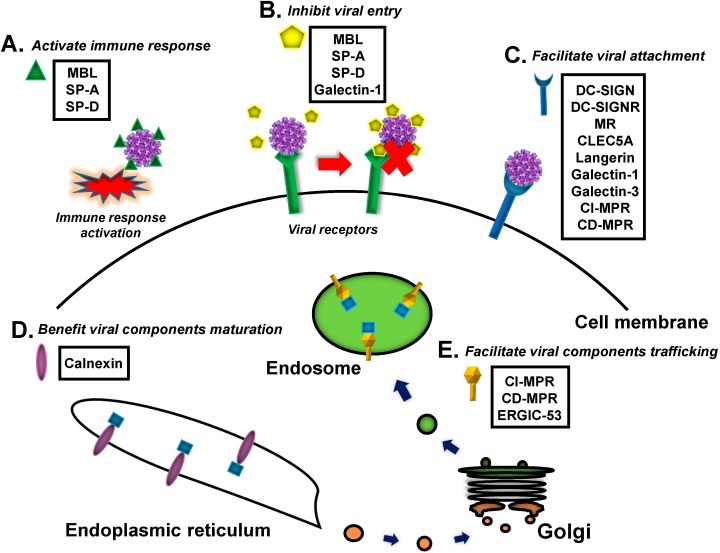
The role of lectins in viral infection. (**A**) Activate immune response; (**B**) Inhibit viral entry; (**C**) Facilitate viral attachment; (**D**) Benefit viral components maturation; (**E**) Facilitate viral components trafficking. The lectins involved in each role are listed in the black box.

The recognition of a specific carbohydrate structure is considered to be a common and effective method for distinguishing between self and non-self factors in animals. Many viral components are highly modified by particular oligosaccharides, and lectins are capable of recognizing viral glycoproteins and thus function in host-virus interactions. In this review, we globally summarize the roles of direct lectin-virus recognition in host immune responses and pathogenesis during viral infections. The viral recognition by some lectins can activate host immunity, resulting in viral elimination; in contrast, other lectins are exploited as susceptibility factors to facilitate viral entry, replication or assembly (Figure 1). Insight into direct lectin-based viral recognition will provide a deep understanding of host-virus interactions.

## 2. Viral Recognition by Mammalian Lectins

Mammalian lectins have been categorized into multiple classes according to the features of their CRDs, as well as their sugar recognition specificity. Some lectins generally play a role outside the cell, whereas others are predominantly intracellular and located on cytoplasmic organelles. Extracellular lectins, including C-type, R-type, I-type/siglecs lectins and galectins, are secreted into the extracellular milieu or are localized to the plasma membrane and are capable of mediating cell adhesion, immune signaling and pattern recognition activities for host-pathogen interactions. However, intracellular lectins, such as the calnexin family, M-type, L-type and P-type lectins, are located in luminal compartments of the secretory pathway and function in the trafficking, sorting and maturation of glycoproteins [3,4,5]. As lectins play diverse roles in mammalian physiological processes, we only focus herein on a portion of lectins that directly interact with viral components and describe their functions in viral propagation and pathogen-host immune responses.

### 2.1. C-Type Lectins

C-type lectins (CTLs) are a large group of proteins in metazoans that were originally named according to their property of Ca^2+^ (C-type)-dependent carbohydrate binding. Sequence and structural comparisons of C-type lectins have suggested that their carbohydrate-binding activity is mediated by a specific CRD that is conserved in a variety of organisms. Although some C-type lectins do not possess carbohydrate-binding activity, all of them show distinct sequence similarity and are believed to descend from a common ancestor during evolution [6,7,8]. To date, C-type lectins have been divided into 14 subgroups according to their domain architecture and the phylogenetic relationship between their CRD sequences [9].

**Figure 2:**
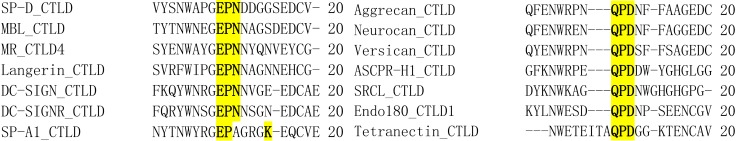
Sequence alignment of carbohydrate-binding motifs. The core sequences of the carbohydrate-binding region of several C-type lectins were aligned using ClustalW2. The E-P-N and Q-P-D motifs are highlighted in yellow. SP-D: Surfactant protein-D; CTLD: C-type lectin domain; MBL: Mannose-binding lectin; MR: Mannose receptor; SP-A1: Surfactant protein-A1; ASCPR-H1: Asialoglycoprotein receptor H1 subunit; SRCL: Scavenger receptor with CTLD.

In general, C-type lectins can be separated into two groups, mannose-binding and galactose-binding C-type lectins, based on the specificity of their carbohydrate-binding activity. The binding specificity of these two groups is mediated by diverse residues flanking the conserved *cis*-proline in the long loop region, in which the sequence of the core motif is E-P-N for mannose-binding and Q-P-D for galactose-binding specificity [10,11]. Previous studies have demonstrated that interchange of the E-P-N and Q-P-D sequences is sufficient to switch the mannose- and galactose-binding specificity (Figure 2) [10]. However, several lectins are exceptions to this rule. For example, surfactant protein A, possessing an E-P-K but not an E-P-N motif, binds to mannose sugars [12,13]. Although human tetranectin contains a galactose-type Q-P-D motif, it is not responsible to the lectin-carbohydrate interaction [14]. Therefore, other determinants, including modifications around the binding sites and stereochemical factors, should be taken into consideration when examining binding specificity [15,16,17]. Robust investigations have shown that multiple mannose-/galactose-binding C-type lectins play important roles in viral infections in mammals.

#### 2.1.1. Mannose-Binding Lectin (MBL)

**Figure 3:**
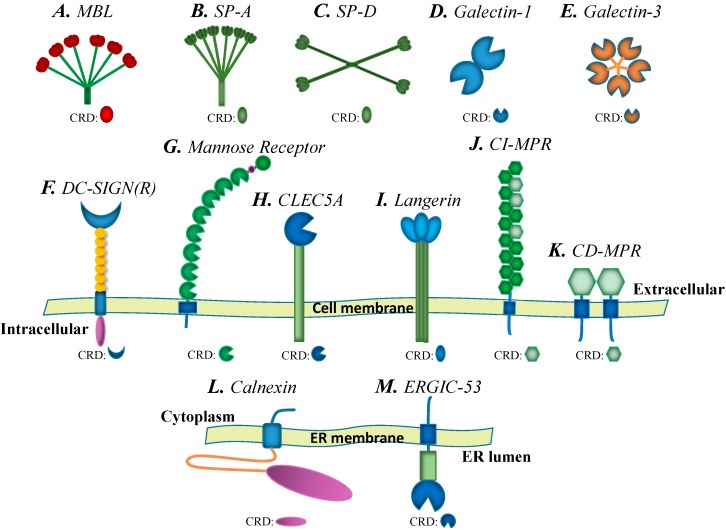
Diagram of lectin structures. (**A**) MBL; (**B**) SP-A; (**C**) SP-D; (**D**) Galectin-1; (**E**) Galectin-3; (**F**) DC-SIGN(R): DC-SIGN and DC-SIGNR; (**G**) Mannose Receptor; (**H**) CLEC5A; (**I**) Langerin; (**J**) CI-MPR; (**K**) CD-MPR; (**L**) Calnexin; (**M**) ERGIC-53.

MBL, one of the most intensively studied lectins, is a member of the collectin family, a subgroup of C-type lectins (Figure 3A). The MBL molecule contains four domains that are standard for collectin family proteins: a cysteine-rich region, a collagen-like domain, a neck region and a carbohydrate recognition domain. The native functional form of MBL is a hexamer; however, although MBL can form several oligomeric forms, the dimers and trimers do not have biological activity, and at least a tetramer form is needed to activate the complement cascade [18]. MBL functions as a soluble pattern recognition receptor in the host complement system and is involved in resistance to many viral infections [19]. The CRDs of MBL multimers recognize carbohydrate patterns on the virus surface, and consequently, the binding of MBL and viral particles results in activation of the lectin pathway of the complement system. MASPs (mannose-binding lectin-associated serine proteases), which are protease zymogens (an inactive form of an enzyme) similar to the C1r and C1s molecules of the classical complement pathway, are activated to cleave complement components C4 and C2 into C4a/C4b and C2a/C2b, respectively. Interactions between C4b and C2b produce the C3 convertase, which continuously activates C3 further downstream in the cascade to eliminate viruses (Figure 1A) [20]. Current investigations have reported a resistance role of MBL in infections of multiple human viruses, including Human immunodeficiency virus (HIV) [21], Hepatitis B virus (HBV) [22,23], Hepatitis C virus (HCV) [24], West Nile virus (WNV) [25] and Dengue virus (DENV) [25]. Specific recognition of these viral particles by MBL is a central event for activation of the lectin-based complement cascade.

The mechanism of viral elimination by the MBL-based complement cascade is still unclear. Unlike bacterial elimination by the complement system, MBL-based complement components do not appear to form a membrane-attacking complex on the viral surface; therefore, viral eradication may not be mediated by known complement mechanisms. Three possible mechanisms have been proposed for MBL-mediated viral elimination. (1) MBL-mediated complement C3/C4 deposition onto the viral surface. Viral neutralization can be processed in cell-free serum and is completely dependent on C3 and C4 activation, but not on C1q and C5, suggesting that neither opsonization nor the classical/alternative complement pathway is sufficient for viral neutralization [25]. (2) MBL can directly neutralize viruses. Pre-incubation of serial concentrations of recombinant MBL with HIV cell-derived living particles was found to dramatically neutralize HIV infection [21,26]. However, other independent investigations have suggested that some primary HIV isolates resist direct neutralization by MBL [27], indicating that the possible neutralizing activity depends highly on the different carbohydrate structures on the surfaces of various viral strains. (3) MBL can block the recognition of viruses and receptors. Dendritic cell-specific intercellular adhesion molecule 3-grabbing nonintegrin (DC-SIGN), which is present on the surface of dendritic cells, functions as a key attachment factor used for the recognition and uptake of multiple viruses [28,29,30,31,32]. A study reported that MBL can prevent interaction between HIV and DC-SIGN, thereby inhibiting the HIV infection of T cells, which is mediated by DC-SIGN [33]. Furthermore, MBL interacts with the viral envelope glycoproteins of Ebola and Marburg viruses (MARV), resulting in the impairment of viral internalization by blocking virus-DC-SIGN interaction (Figure 1B) [34].

#### 2.1.2. Surfactant Proteins (SPs)

Two soluble collectins, designated SP-A and SP-D (Figure 3B,C), have been found to be involved in the recognition of viral particles for limiting infection in humans. SP-A is produced within the respiratory tract, gastrointestinal tract, and possibly other sites; conversely, SP-D is primarily synthesized in the respiratory tract [35]. These factors are constitutively secreted into the lungs by alveolar type II cells, unciliated bronchial epithelial cells and other mucosal tissue cells [36,37]. The specific location of surfactant proteins suggests a defensive role against viral invasion of the respiratory system.

Both SP-A and SP-D interact with different strains of Influenza A virus (IAV) via glycosylated hemagglutinin (HA) and neuraminidase (NA) on the viral surfaces. The binding of IAV to SP-A leads to agglutination of the virions, inhibition of IAV infectivity and dissemination and also facilitates clearance by macrophages and neutrophils (Figure 1A,B) [38]. SP-D binds to IAVs and thereby inhibits virus attachment and entry by viral aggregation (Figure 1B) [39,40,41,42,43] and also controls IAV infection in human by activating neutrophil chemoattraction (Figure 1A) [44,45].

Respiratory syncytial virus (RSV) infects humans via the respiratory tract. SP-A has been reported to bind fusion (F) and adherence (G) glycoproteins on the surfaces of RSV virions, resulting in opsonization to reduce infection by enhancing viral uptake by peripheral blood mononuclear cells (PBMCs) and alveolar macrophages (Figure 1A) [46,47]. SP-D also directly interacts with RSV surface G protein to modulate host immune responses to control RSV infection [48].

#### 2.1.3. DC-SIGN, L-SIGN and the Mannose Receptor (MR)

DC-SIGN (CD209) and its homolog L-SIGN (also called DC-SIGNR, CD209L) are one of the most investigated C-type lectins involved in viral infection (Figure 3F). Unlike the main role of collectins in host defense, DC-SIGN and L-SIGN have been widely reported to be susceptibility factors that facilitate viral entry into host immune cells [28,31,32,49]. Both DC-SIGN and L-SIGN are trans-membrane proteins that are composed of a short cytoplasmic tail, which is responsible for signaling and internalization, a transmembrane region, a neck domain, which consists of eight repeat regions of 23 amino acids, and a carbohydrate recognition domain [50].

The roles of DC-SIGN/L-SIGN in viral infections have been summarized and reviewed previously [50,51]. Studies have shown that DC-SIGN/L-SIGN are capable of binding to the surface proteins of HIV [28], cytomegalovirus (CMV) [30], DENV [32], WNV [52,53], Severe acute respiratory syndrome coronavirus (SARS-CoV) [54,55,56], HCV [57,58], Ebola virus [29] and MARV [54] and consequently facilitating viral entry (Figure 1C). Differential glycosylation patterns of viral surface proteins strongly influence the efficiency of viral recognition by DC-SIGN/L-SIGN [59,60]. For example, only mannosylated Envelope (E) glycoproteins on DENV, but not E proteins with complex glycosylation, have been shown to interact with DC-SIGN-expressing cells [60].

The mannose receptor (MR, CD206) is another C-type lectin that functions as a viral recognition receptor on the cell membrane (Figure 3G). MR is mainly expressed in multiple immune cells, including macrophages and dendritic cells, and is a key susceptibility factor for DENV infection of human macrophages. Binding of MR to DENV E glycoproteins enhances viral attachment, thus facilitating DENV internalization into macrophages, and deglycosylation of the DENV E glycoprotein enables the abrogation of this binding, and DENV infection of primary human macrophages can be blocked by anti-MR antibodies [61]. Moreover, the interaction between MR and HBV surface antigen (HBsAg) enhances viral uptake by dendritic cells (DCs), resulting in the impairment of DC function and the ineffective antiviral response of chronic HBV [62]. The recognition of viral surface glycoproteins by MR is also beneficial to influenza virus [63,64] and HIV [65] invasion into host cells (Figure 1C). Overall, DC-SIGN/L-SIGN and MR function as receptors/attachment factors for viral entry into particular cell types.

#### 2.1.4. C-Type Lectin Domain Family 5, Member A (CLEC5A)

CLEC5A/MDL-1 (myeloid DAP12-associating lectin) is a C-type lectin associated with DAP12 (12-kDa DNAX-activating protein) on myeloid cells such as monocytes, macrophages and neutrophils (Figure 3H) [66]. A recent study has found that CLEC5A binds to dengue glycoproteins. However, in contrast to other C-type lectin receptors, the association between CLEC5A and DENV does not result in viral entry, but rather induces DAP12-mediated immune signaling to stimulate the release of pro-inflammatory cytokines that potentially contribute to the pathogenesis of dengue hemorrhagic fever [67,68]. CLEC5A also directly interacts with Japanese encephalitis virus (JEV) to induce DAP12 phosphorylation in macrophages and therefore plays a role in JEV-induced neuro-inflammation and lethality (Figure 1C) [69]. The blocking of CLEC5A in mice can significantly reduce the infiltration of JEV-harboring leukocytes into the central nervous system, thus attenuating neuro-inflammation and protecting the animals from JEV-induced lethality [69]. The discovery of a role of CLEC5A in flaviviral pathogenesis suggests that the extracellular CRD modules are generally responsible for the recognition of viral glycoproteins; nonetheless, the intracellular modules determine the role of C-type lectins in viral infection.

#### 2.1.5. Langerin

Langerin (also known as CD207), containing a single Ca^2+^-dependent CRD domain, is a type II transmembrane C-type lectin that is specifically expressed on Langerhans cells (Figure 3I). The physiological function of langerin is to trigger the cellular membrane superimposition and zippering that benefit Birbeck granule (BG) formation [70]. Langerin is capable of directly binding to HIV-1 envelope protein gp120 and thus serves as a potential receptor for HIV-1 infection in Langerhans cells (Figure 1C) [71,72]. However, a recent study reported that langerin is a natural barrier for HIV-1 transmission among Langerhans cells. Langerin is capable of directly capturing HIV-1 and sequentially degrading it in BGs to promote T cell elimination of HIV-1 infection [73], suggesting that langerin plays a pleiotropic role in HIV infection. Furthermore, langerin functions as an attachment factor to facilitate Measles virus (MV) infection in Langerhans cells [74].

A large number of host proteins are abundantly glycosylated. Therefore, microbial recognition by C-type lectins relies on the mechanism for distinguishing carbohydrate structures between self and non-self, and the C-type lectin structure largely influences binding avidity and selectivity in the recognition of self and non-self carbohydrate structures [75,76]. Based on the X-ray crystal structures of mannose-binding lectin (MBL), the MBL CRD sites in the trimer form are too far apart to spatially interact efficiently with common mammalian high-mannose oligosaccharides. However, the dense and repeated arrays of carbohydrates present on the microbial surface can span the distance between the binding sites in MBL, resulting in highly avid multivalent interaction [75,77]. Moreover, the number of CRDs is another determinant for the avidity and strength of differential binding by C-type lectins. The eight different C-type CRDs of MR contribute to its high binding affinity for single sugars, even though each individual CRD motif only displays weak affinity. The CRD domain organization also confers MR with the ability to recognize the wide range of different carbohydrates found on the pathogen surface and to distinguish between self and foreign glycoproteins [75].

### 2.2. Galectins

Galectins are a group of secreted proteins that associate with specific cell surface glycans containing beta-galactosides (Figure 3D,E) [78]. Although mammalian galectins lack conventional signal sequences, they reach the cell surface via a particular mechanism. Galectins accumulate directly beneath the plasma membrane and are subsequently involved in the establishment of membrane-bound vesicles that pinch off before release outside the cell; galectins then bind to glycoconjugates on the plasma membrane or remain in the extracellular matrix [79,80,81]. Fifteen galectins have been identified in mammals and are categorized into three structural forms: dimeric, tandem or chimeric. Dimeric galectins, also called prototypical galectins, are homodimers and include galectin-1, -2, -5, -7, -10, -11, -13, -14 and -15. Tandem galectins contain at least two distinct CRDs within one polypeptide and include galectin subtype-4, -5, -8, -9 and -12. Galectin-3 is specific to mammals, has one CRD and a long non-lectin domain, and exists in either a monomeric form or a multivalent complex associated via the non-lectin domains of monomers [82]; this property allows galectin-3 to effectively bridge different ligands to form adhesive networks. Current investigations have indicated that direct recognition between galectin-1/-3 and viral-surface glycoproteins is important for host-virus interaction.

Galectin-1 is secreted by immune cells, such as T helper cells in the thymus, or by stromal cells surrounding B cells and is abundant in muscle, neurons and kidneys [83]. Because of its particular binding specificity for galactosides, galectin-1 recognizes the surface envelope proteins of many human viruses and therefore is involved in viral infection. Galectin-1 binds to NiV-F, a viral envelope glycoprotein of Nipah virus (NiV), to reduce the NiV-F-mediated fusion of endothelial cells and thereby inhibit NiV-induced syncytium formation [84]. Galectin-1 directly interacts with the envelope glycoproteins of Influenza A/WSN/33 virus and inhibits its hemagglutination activity, resulting in the reduction of influenza virus infectivity (Figure 1B) [85]. However, galectin-1 has also been reported to be a susceptibility factor for viral entry. HIV-1 exploits galectin-1 to enhance gp120-CD4 interaction, leading to faster viral entry and more robust viral replication (Figure 1C) [86,87,88,89]. In addition to galectin-1, the role of galectin-3 in viral infection has been elucidated by several studies. Galectin-3 has been shown to interact with Herpes simplex virus-1 (HSV-1). RNAi-mediated knockdown of galectin-3 in human corneal keratinocytes significantly impaired HSV-1 infection, suggesting that HSV-1 exploits galectin-3 to enhance its attachment to host cells [90]. Recently, proteomic-based studies have identified galectin-3 as a host-binding partner of parvovirus Minute virus of mice (MVM). The authors proposed that galectin-3 binding facilitates the access of MVM to its receptor(s) at the plasma membrane and thus promotes MVM endocytosis (Figure 1C) [91]. The above-mentioned evidence indicates a pleiotropic role of galectins during viral infections.

### 2.3. Calnexin Family

The endoplasmic reticulum (ER) of mammalian cells contains molecular chaperones and foldases, which are required for forming the active structures of newly synthesized peptides and thus serve as components of the ER quality control system. The ER-resident chaperones include BiP, calnexin (Figure 3L) and calreticulin (a calnexin-like soluble form without the transmembrane region) [92,93]. Both calnexin and calreticulin are lectin-like, membrane-bound molecular chaperones that associate with newly synthesized proteins in the ER. In addition, several studies have indicated that calnexin and calreticulin preferentially interact with glycoproteins that carry monoglucosylated N-linked oligosaccharides [94,95,96,97].

**Figure 4:**
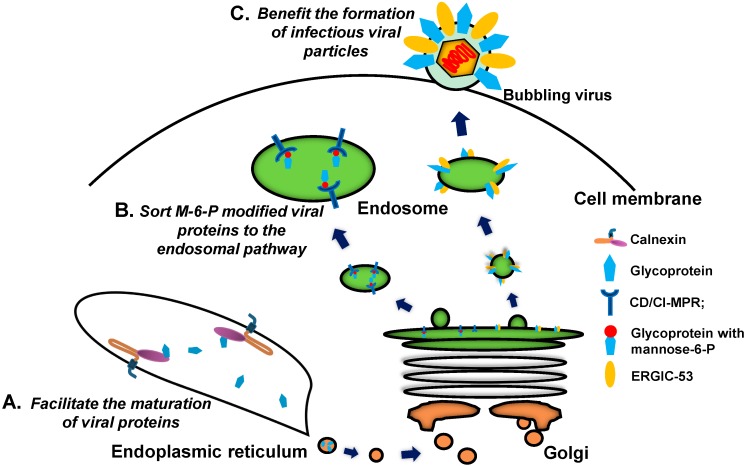
The roles of intracellular lectins in the cytosolic pathway. (**A**) Facilitate the maturation of viral proteins; (**B**) Sort M-6-P modified viral proteins to the endosomal pathway; (**C**) Benefit the formation of infectious viral particles.

The maturation of virus-encoded proteins occurs in the ER, and calnexin family proteins have been shown to transiently interact with multiple viral proteins that consequently undergo rapid maturation (Figure 1D and Figure 4A). Both calnexin and calreticulin can transiently interface with envelope glycoproteins F and HN of Sendai virus (SeV) [98] and glycoproteins G1/G2 of Uukuniemi virus (UUKV) (*Bunyaviridae Family*) [99] to facilitate the rapid maturation of these proteins. During SARS-CoV infection, maturation of the viral S protein due to its interaction with calnexin is essential for the formation of infective virions [100]. Calnexin/calreticulin also plays a role in the assembly and secretion of HBV Middle (M) envelope protein [101], HIV-1 envelope protein gp160 [102] and gp120 [103]. Furthermore, during the rotavirus life cycle, calnexin binds to the ER-associated viral transmembrane protein NSP4, a nonstructural glycoprotein that acts as a toxin capable of inducing diarrhea in animals [104,105,106]. Calnexin/calreticulin is also associated with the glycosylation of Hantaan virus (HTNV, also known as hantavirus) envelope proteins Gn and Gc and plays a crucial role in the folding of HTNV glycoproteins with a high content of high-mannose oligosaccharides [107]. Accumulated evidence suggests that the lectin-like calnexin proteins interact with viral components to largely facilitate viral assembly and protein maturation.

### 2.4. P-Type Lectins/Mannose 6-Phosphate Receptors (MPRs)

The P-type lectin/mannose 6-phosphate receptors (MPRs) are transmembrane glycoproteins that target lysosomal enzymes located in either intracellular organelles or the plasma membrane (Figure 3J,K). MPRs can bind newly synthesized lysosomal hydrolases in the trans-Golgi network (TGN) and deliver them to pre-lysosomal compartments. The MPR CRD was originally identified in two types of proteins, cation-independent and cation-dependent mannose 6-phosphate receptors (CI-MPR and CD-MPR, respectively), both of which recognize mannose-6-phosphate (M-6-P) to identify and route lysosomal enzymes to the lysosomal compartment [108].

A previous study demonstrated that Human herpes simplex virus (HSV) glycoprotein D (gD) binds to both CI-MPR and CD-MPR. These MPRs sort glycoproteins modified with M-6-P to lysosomes in the trans-Golgi compartment and divert them to the endosomal pathway (Figure 1E and Figure 4B) [109,110]. MPRs were also found on the surfaces of mammalian cells as serving as putative cellular receptors for HSV entry and cell-cell viral spread; furthermore, chemo- or immuno-blocking MPRs was shown to inhibit HSV entry and the production of HSV plaques in monkey cells (Figure 1C). Mouse cells lacking both CI-MPR and CD-MPR remain sensitive to HSV infection [110], suggesting that the expression of MPRs is not essential for HSV invasion. Varicella zoster virus (VZV) is known as a highly infectious human pathogen, and multiple VZV envelope glycoproteins are modified by M-6-P; therefore, CI-MPRs appear to be important for VZV infection. Intracellular CI-MPR contributes to the transport of enveloped VZV to late endosomes, and the plasmalemmal form is necessary for cellular entry through cell-free VZV particles [111].

### 2.5. L-Type Lectins

L-type lectins are widely distributed in plants and animals. Animal L-type lectins are intracellular luminal proteins that are involved in protein sorting in the luminal ER-Golgi compartments of animal cells. There are four L-type lectins in mammals: ERGIC-53 (Figure 3M), ERGL, VIP36, and VIPL [112,113]. A recent study has shown that intracellular cargo receptor ERGIC-53 interacts with the glycoproteins of arenavirus, hantavirus, coronavirus, orthomyxovirus, and filovirus particles. ERGIC-53 is also essential for the propagation of arenavirus, coronavirus, and filovirus; in the absence of ERGIC-53, viral particles can be formed but are noninfectious (Figure 1E and Figure 4C) [114].

In addition to the above-mentioned mammalian lectins, there are some other lectin classes in animals, e.g., M-type, R-type, I-type, Chitinase-like, F-box lectins and intelectins. As we have not found reports on direct interactions between these lectins and viral components, we cannot determine the role of these lectins in viral infection based on the current knowledge.

## 3. Arthropod Lectins and Viral Infections

Lectin CRDs are conserved throughout evolution, and many lectin homologues have been identified and reported in invertebrates. A homolog of galectin plays a role in opsonization for bacterial clearance in *Marsupenaeus japonicus* [115]. Similarly, a galectin-like factor is expressed on the surface of oyster hemocytes and plays a role in oyster physiology through the recognition of oligosaccharides [116,117]. Several proteins identified in *Crassostrea hongkongensis* [118], *Venerupis philippinarum* [119] and *Trypanosoma cruzi* [120] have been categorized as homologs of mammalian I-type lectins/Siglecs with high sialic acid-binding activity. In arthropods, multiple lectins identified in shrimp, such as L-type, P-type/MPRs, M-type, and calnexin family factors, have been proposed to be important in shrimp innate immunity [121]. Many C-type lectin homologues in *Aedes* and *Anopheles* mosquitoes have been found to be involved in insect immune responses and pathogenesis [122,123,124].

The current investigations of the immune roles of arthropod lectins mainly focus on their anti-bacterial or anti-parasite functions, including microorganism-induced lectin up-regulation, lectin-mediated microorganism recognition and opsonization [125,126]. However, little is known about the molecular details of lectins in arthropod immunity and pathogenesis, especially with regard to the function in arthropod-virus interactions. A recent study on C-type lectins in *Aedes aegypti* initially assessed lectin functions in viral infections of arthropods. Tens of C-type lectins were identified in *Aedes* [122,123] and *Anopheles* [124] mosquitoes, and most are soluble forms [127]. Previous studies have shown that an *Aedes aegypti* C-type lectin, mosquito galactose-specific C-type lectin-1 (*mosGCTL-1*), interacts with WNV in a calcium-dependent manner to form a mosGCTL-virus complex. This complex consequently interacts with mosquito protein tyrosine phosphatase-1 (*mosPTP-1*), a mosquito homolog of human CD45 in *A. aegypti*, to enable viral attachment to the plasma membrane and enhance viral entry. *In vivo* experiments showed that mosGCTL-1 and mosPTP-1 function as part of the same pathway and are critical for WNV infection of mosquitoes [127]. Further investigations identified that another 9 *mosGCTL* paralogs facilitate dengue infection of mosquitoes. These mosGCTLs interact with DENV-2 surface E protein and virions, functioning as susceptibility factors for dengue viral entry into mosquito cells. However, *mosPTP-1* did not influence dengue infection in mosquitoes, suggesting that other membrane receptors may recruit the DENV-mosGCTL complex onto the cell membrane for viral entry [122].

In agreement with the findings in mosquitoes, a recent study has identified a C-type lectin in the shrimp *Marsupenaeus japonicus* that interacts with an envelope protein of White spot syndrome virus (WSSV) and consequently associates with a cell-surface calreticulin, which serves as a membrane receptor that facilitates viral entry in a cholesterol-dependent manner [128]. The study therefore suggested that C-type lectins might play a broad role in expediting many viral infections of arthropods. The role might not be limited to WNV/DENV in mosquito and WSSV in shrimp but might extend to other virus infections in arthropods.

## 4. Anti-Viral Drug and Vaccine Targeting of Lectins

Lectins are potential targets for the development of antiviral drugs and vaccines. Such lectin-based antiviral strategies are divided into two parts: (1) lectin-based immune activation and (2) blockade of lectin receptors against viral entry [129]. Many envelope viruses are protected by their dense carbohydrate shield against efficient recognition and persistent neutralization by the host immune system. Various natural and synthetic carbohydrate-binding agents have been screened to refine candidates that can reinforce the recognition of specific pathogens, enhance the cascade amplification of the innate immune response and interrupt virus attachment to receptors. In fact, lectins have been considered as drug targets for many years. Several heterologous lectins derived from various organisms have been already selected and introduced into pre-clinic trials for HIV therapy, including SVN (scytovirin), a 9.7-kD lectin isolated from aqueous extracts of the cyanobacterium *Scytonema varium* [130], and UDA (stinging nettle lectin), a 8.5-kD plant lectin isolated from *Urtica dioica* [131,132]. Furthermore, the combined usage of UDA with HHA (Amaryllis lectin, from a *Hippeastrum* hybrid) and GNA (Snowdrop lectin from *Galanthus nivalis*), another two carbohydrate-binding agents, showed broad anti-viral activity against four serotypes of DENV in monocyte-derived dendritic cells by preventing virus attachment [133]. Additionally, an interesting monoclonal antibody, 2G12, which interacts with specific, highly conserved glycosylation sites on HIV envelop protein gp120 shows a broad anti-HIV neutralizing activity. The mechanism of this antibody specifically targeting N-linked glycans is very similar to that of lectins [134,135,136].

With regard to arthropod-borne viruses, vector ligands that interact with pathogens are ideal targets for interfering with the successful acquisition of the virus from the vertebrate host. Due to the importance of C-type lectins in dengue infection of mosquitoes, these lectin factors may be proposed as targets for the development of vaccines or antiviral drugs. Studies show that treatment with mosGCTLs antisera dramatically interrupted DENV-2 infection of mosquitoes through blood feeding. Therefore, the humoral response against mosGCTLs in mammals could feasibly impair dengue infection of mosquitoes. The approach to blocking mosquito C-type lectins may direct a future avenue for the development of a transmission-blocking vaccine that interrupts the mosquito-borne viral life cycle and reduces disease burden [122].

**Table 2:** The role of lectins in viral infection.

Species	Lectin Family	Name	Location	Virus	Target Protein	Function	Reference
Mammal	C-type lectins	MBL	Extracellular	HIV	gp120	Inhibit viral infection	[21]
				HBV	HBsAg	Inhibit viral infection	[22,23]
				HCV	Envelope glycoproteins	Inhibit viral infection	[24]
				WNV	E protein	Inhibit viral infection	[25]
				DENV	E protein	Inhibit viral infection	[25]
				Ebola virus	Envelope glycoproteins	Inhibit viral infection	[34]
				MARV	Envelope glycoproteins	Inhibit viral infection	[34]
		SP-A	Extracellular	IAV	HA and NA	Inhibit viral infection	[38]
				RSV	F and G protein	Inhibit viral infection	[46,47]
		SP-D	Extracellular	IAV	HA	Inhibit viral infection	[39,40,41,42,43]
				RSV	G protein	Inhibit viral infection	[48]
		DC-SIGN(R)	Transmembrane	HIV	gp120	Facilitate viral infection	[28]
				CMV	Envelope glycoproteins	Facilitate viral infection	[30]
				DENV	E protein	Facilitate viral infection	[32]
				WNV	E or prM protein	Facilitate viral infection	[52,53]
				MARV	GPs	Facilitate viral infection	[54]
				HCV	E2 protein	Facilitate viral infection	[57,58]
				Ebola virus	GP1 subunit	Facilitate viral infection	[29]
				SARS-CoV	S protein	Facilitate viral infection	[54,55,56]
		MR	Transmembrane	DENV	E protein	Facilitate viral infection	[61]
				HBV	HBsAg	Facilitate viral infection	[62]
				IAV	HA or NA	Facilitate viral infection	[63,64]
				HIV	gp120	Facilitate viral infection	[65]
		CLEC5A	Transmembrane	DENV	E protein	Facilitate viral infection	[67,68]
				JEV	E protein	Facilitate viral infection	[69]
		Langerin	Transmembrane	HIV	gp120	Facilitate viral infection	[71,72]
				HIV	gp120	Inhibit viral infection	[73]
				MV	F and H protein	Facilitate viral infection	[74]
Mammal	Galectins	Galectin-1	Secrete/Membrane	NiV	F proteins	Inhibit viral infection	[84]
				IAV	Envelope glycoproteins	Inhibit viral infection	[85]
				HIV	gp120	Facilitate viral infection	[86,87,88,89]
		Galectin-3	Secrete/Membrane	HSV	Envelope glycoproteins	Facilitate viral infection	[90]
				MVM	Capsid protein	Facilitate viral infection	[91]
	Calnexins	Calnexin/calreticulin	Intracellular	SeV	F and HN protein	Facilitate viral infection	[98]
				UUKV	G1/G2 protein	Facilitate viral infection	[99]
				SARS-CoV	S protein	Facilitate viral infection	[100]
				HBV	M protein	Facilitate viral infection	[101]
				HIV	gp160 and gp120	Facilitate viral infection	[102,103]
				Rotavirus	NSP4	Facilitate viral infection	[104,105,106]
				Hantavirus	Gn and Gc	Facilitate viral infection	[107]
	P-type lectins/MPRs	CI/CD-MPR	Transmembrane	HSV	gD	Facilitate viral infection	[109,110]
				VZV	Envelope glycoproteins	Facilitate viral infection	[111]
			Intracellular	HSV	gD	Facilitate viral infection	[110]
				VZV	gB, gE, gH and gI	Facilitate viral infection	[111]
	L-type lectins	ERGIC-53	Intracellular	Arenavirus, Hantavirus, Coronavirus, Filovirus, Orthomyxovirus	GPs	Facilitate viral infection	[114]
Arthropod	C-type lectins	mosGCTLs	Extracellular	WNV	E protein	Facilitate viral infection	[127]
				DENV	E protein	Facilitate viral infection	[122]
		MjsvCL	Extracellular	WSSV	Protein 28	Facilitate viral infection	[128]

Notes: MBL: Mannose binding lectin; SP-A, SP-D: Surfactant proteins A, D; DC-SIGN(R): DC-SIGN and L-SIGN; MR: Mannose receptor; CLEC5A: C-type lectin domain family 5, member A; CI/CD-MPR: cation-independent/cation-dependent mannose 6-phosphate receptor; ERGIC-53: (ER)Golgi intermediate compartment 53 kDa protein; mosGCTLs: *Aedes aegypti* C-type lectins; MjsvCL: *Marsupenaeus japonicusstomach* stomach virus–associated C-type lectin; HIV: Human immunodeficiency virus; HBV: Hepatitis B virus; HCV: Hepatitis C virus; WNV: West Nile virus; DENV: Dengue virus; MARV: Marburg virus; IAV: Influenza A virus; RSV: Respiratory syncytial virus; CMV: Cytomegalovirus; SARS-CoV: Severe acute respiratory syndrome coronavirus; JEV: Japanese encephalitis virus; MV: Measles virus; NiV: Nipah virus; HSV: Herpes simplex virus; MVM: Minute virus of mice; SeV: Sendai virus; UUKV: Uukuniemi virus; VZV: Varicella zoster virus; WSSV: White spot syndrome virus; HA: Oligosaccharides hemagglutinin molecules; NA: Neuraminidase; GPs: Surface glycoproteins; NSP4: None structure protein 4; gD: glycoprotein D; gB, gE, gH and gI: glycoprotein B, E, H, I.

## 5. Conclusions

Lectins comprise highly diverse proteins with different carbohydrate recognition activities and play pleiotropic roles in the immune responses and pathogenesis of many viral infections (summarized in Table 2). The interaction between lectins and viral glycoproteins may lead to the three following consequences: (1) lectins, such as MBL and SPs, function as pattern recognition molecules that bind a repertoire of viruses and activate antiviral immune responses; (2) lectins are employed as attachment factors that recruit viral particles to the cell membrane to enhance viral entry, e.g., some mammalian lectins (DC-SIGN, L-SIGN, MR and MPRs) or their homologs in arthropods (mosGCTLs); and (3) some intracellular lectins, such as calnexin and ERGIC-53, function as susceptibility factors associated with virus-encoded proteins to facilitate viral replication or assembly (please refer to Figure 1 and Figure 4).

Interestingly, the same lectin may show opposing roles in different virus infections. For example, galectin-1 binds to NiV to inhibit syncytium formation and recognizes IAV to reduce its infectivity [84,85]; however, galectin-1 was also reported to be a susceptibility factor that enhanced gp120-CD4 interactions, thus facilitating HIV entry [86,87,88,89]. The current accumulated knowledge indicates that lectins are crucial host factors with complex and profound roles in the process of viral infection.
